# Acute Myocarditis in a Patient with Newly Diagnosed Granulomatosis with Polyangiitis

**DOI:** 10.1155/2015/134529

**Published:** 2015-12-07

**Authors:** Anne Munch, Jens Sundbøll, Søren Høyer, Manan Pareek

**Affiliations:** ^1^Department of Oncology, Aarhus University, Nørrebrogade 44, 8000 Aarhus C, Denmark; ^2^Department of Cardiology, Aarhus University Hospital, Palle Juul Jensens Boulevard 99, 8200 Aarhus N, Denmark; ^3^Institute of Pathology, Aarhus University Hospital, Nørrebrogade 44, 8000 Aarhus C, Denmark; ^4^The Cardiovascular and Metabolic Preventive Clinic, Department of Endocrinology, Centre for Individualized Medicine in Arterial Diseases, Odense University Hospital, 5000 Odense C, Denmark

## Abstract

A 22-year-old woman recently diagnosed with granulomatosis with polyangiitis (GPA) was admitted to the department of cardiology due to chest pain and shortness of breath. The ECG showed widespread mild PR-segment depression, upwardly convex ST-segment elevation, and T-wave inversion. The troponin T level was elevated at 550 ng/L. Transthoracic echocardiography showed basal inferoseptal thinning and hypokinesis, mild pericardial effusion, and an overall preserved left ventricular ejection fraction of 55%. Global longitudinal strain, however, was clearly reduced. Cardiac magnetic resonance imaging (MRI) showed findings consistent with myocarditis but the etiology of the apical hypokinesis could not be determined with certainty and may well have been due to a myocardial infarction, a notion supported by a coronary angiogram displaying slow flow in the territory of the left anterior descending artery. Finally, an endomyocardial biopsy confirmed the diagnosis of myocarditis. The cardiac symptoms subsided upon treatment with high-dose prednisolone and rituximab.

## 1. Introduction

GPA, formerly known as Wegener's granulomatosis, is a primary systemic small-vessel vasculitis, which typically produces granulomatous inflammation of the upper and lower airways, necrotizing glomerulonephritis in the kidneys, and is associated with a cytoplasmic pattern of antineutrophil cytoplasmic antibodies (cANCA), with specificity against proteinase-3 (PR3). Although clinically overt cardiac involvement in GPA is relatively rare, it is important to recognize, as adjunctive immunosuppression may be indicated. Furthermore, it is essential to determine the nature of the cardiac condition to ensure appropriately targeted treatment [1–3].

## 2. Case Presentation

A 22-year-old woman was admitted to the department of cardiology due to episodic pressure-like chest pain and shortness of breath. She had received a diagnosis of GPA only a few days earlier, based on cutaneous vasculitis and neuropathy in the lower legs, sinusitis, pulmonary nodules, bilateral wrist arthritis, a markedly elevated PR3-ANCA level > 100 IU/mL (normal range 0–10 IU/mL), and histological findings from the nasal mucosa and lungs supportive of the disease. Vital parameters recorded at admission included a normal blood pressure at 116/83 mm Hg, heart rate at 95 beats per minute, a respiratory rate of 20 breaths per minute, and SpO2 at 97%.

**Figure 1:**
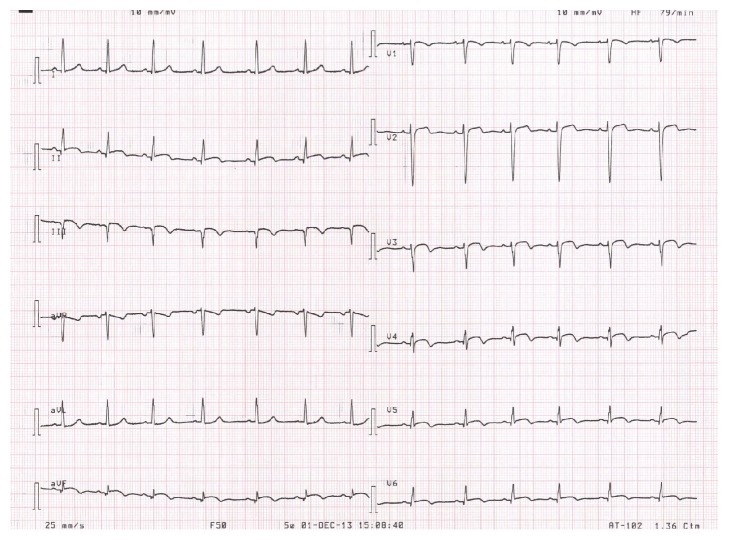
Upward convex ST-segment elevation and T-wave inversion in inferior and precordial leads suggestive of acute ischaemia and mild widespread PR-segment depression suggestive of pericarditis (although nearly subsided in this ECG).

**Figure 2:**
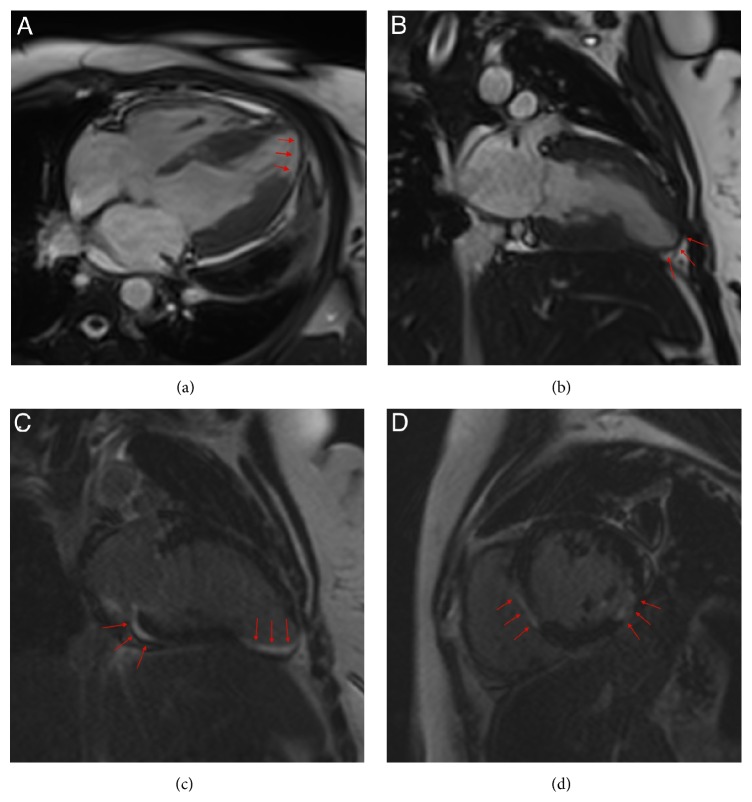
MRI scan with apical thinning of the myocardium (arrows) in 4-chamber view (a) and 2-chamber view (b). Late gadolinium enhancement (arrows) of the apical and inferobasal segments in 2-chamber view (c) and of the inferolateral and septal segments in short axis view (d).

**Figure 3:**
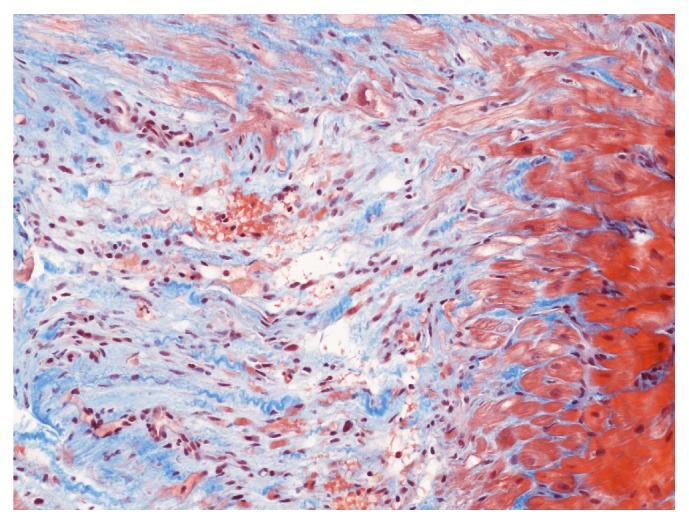
Endomyocardial biopsy showing a large collagenous scar, including several degenerated myocytes. There is a marked vascular proliferation and scanty nongranulomatous chronic inflammation. There is no evidence of vasculitis (Masson Trichrome).

An ECG recorded at admission showed widespread mild PR-segment depression consistent with pericarditis and upward convex ST-segment elevation and T-wave inversion, somewhat more indicative of acute myocardial ischaemia (Figure 1). The troponin T level was elevated at 550 ng/L (normal range 0–13 ng/L), and transthoracic echocardiography showed basal inferoseptal thinning, hypokinesis, and minimal pericardial effusion. The left ventricular ejection fraction was overall preserved at 55%; however, the longitudinal function was clearly reduced as evaluated by both Doppler-based tissue velocity tracking and speckle-tracking based midwall global longitudinal strain. Cardiac magnetic resonance imaging (MRI) showed findings consistent with myocarditis with myocardial hyperaemia and capillary leak in the basal inferoseptal region (Figure 2). Moreover, thinning as well as late gadolinium enhancement was found apically, indicative of transmural infarction. Coronary angiography demonstrated slow flow in the distal LAD but no culprit lesion. Finally, an endomyocardial biopsy from the right ventricle showed fibrosis, degenerated myocytes, and lymphocyte infiltration consistent with active myocarditis (Figure 3).

The patient's GPA was treated with a standard induction regimen of high-dose prednisolone. This was supplemented with rituximab. Shortly after treatment initiation, the symptoms subsided, and troponin T levels declined.

The patient was discharged after a couple of weeks and was followed up in the outpatient clinic one month later at which point she was in clinical and serological remission and no longer had any symptoms consistent with cardiac disease. The ECG revealed persistent, albeit mild, ST-segment elevation in the lateral leads, and the echocardiogram was normal except for persistent apical hypokinesis. At follow-up one year later, the ECG had normalized, and the echocardiogram was unaltered. Therefore, repeat cardiac MRI was not performed.

## 3. Differential Diagnoses

GPA should be considered as a differential diagnosis, whenever a patient presents with multiple organ involvement. The diagnosis is primarily based on characteristic clinical features, specific organ involvement, and histological findings. In this particular case, the findings on cardiac MRI could themselves have warranted a differential diagnosis of sarcoidosis; however, the patient had very recently received a histologically confirmed diagnosis of GPA.

Pulmonary embolism and acute coronary syndrome were suspected at admission, since the patient presented with acute dyspnea, chest pain, and moderately elevated heart rate. The initial suspicion of acute coronary syndrome was supported by the ECG changes (ST-segment elevations) and the elevated troponin T levels. However, as the troponin T levels in the acute phase were fixed at around 550 ng/L without significant fluctuations, and echocardiography did not show signs of right ventricular dysfunction or elevated pulmonary artery pressure, a diagnosis of myocarditis was more plausible [4].

## 4. Discussion

This case report describes a case of GPA with clinically overt cardiac involvement. The diagnosis of myocarditis was supported by complementary imaging modalities and histology. Although we cannot be entirely certain that a causal relationship exists, the close temporal association between the diagnosis of GPA and the onset of cardiac symptoms in an otherwise healthy 22-year-old woman suggests that GPA was responsible for the histologically verified myocarditis. Although the nature of the apical lesion could not be completely determined, we speculate that the active myocarditis somehow resulted in decreased flow in the terminal part of the left anterior descending artery, causing transmural infarction in the most distal part of its supplied territory, that is, the inferior part of the apex.

GPA is a systemic autoimmune disease of unknown etiology. The clinical presentation may vary, but its hallmark features include necrotizing vasculitis in small- and medium-sized blood vessels and granuloma formation, primarily in the respiratory tract and kidneys. Clinically evident cardiac manifestations are rare, but subclinical cardiac involvement may occur in up to 90% of cases, depending on case selection and diagnostic methods. In addition, previous studies have indicated that cardiac involvement may be associated with initial treatment resistance, increased risk of disease relapse, and increased mortality [2, 3, 5].

In symptomatic patients, pericarditis is the most common finding, but coronary artery disease, cardiomyopathy/myocarditis, valvulitis, endocarditis, and conduction abnormalities may be seen as well [6]. Only few reports exist on myocarditis caused by GPA [7]. In these cases, myocardial involvement was most often focal with the basal part of the septum as predilection site, which was in accordance with the findings in our case.

Our case report underscores the fact that GPA truly is a systemic condition that can involve almost every organ in the body. Symptoms suggestive of cardiac disease must entail thorough investigations before cardiac involvement can be ruled out. In the present case, the presence of cardiac involvement was assessed using an integral approach with several complementary imaging modalities, including echocardiography, MRI, and coronary angiography. Cardiac MRI was the most comprehensive imaging modality and has emerged as a leading technique in the noninvasive diagnosis of myocarditis, as it allows for a safe and reproducible description of affected sites, quantification of ventricular volumes, and function and can assess myocardial morphology as well as identify ongoing myocarditis, which is useful not only in the diagnostic process, but also in the follow-up of the patient [8, 9]. However, despite its invasive nature and limited sensitivity, endomyocardial biopsy is still considered the gold standard for the diagnosis of myocarditis caused by GPA and was therefore also performed (Figure 3) [10].

Cyclophosphamide and corticosteroids constitute the classical induction regimen in the treatment of GPA and most often result in clinical remission after 6 months of therapy [11]. However, rituximab is an effective alternative to cyclophosphamide, especially in patients with concerns about fertility or a high risk of malignancy. In this case, initial therapy with rituximab instead of cyclophosphamide was chosen having taken into consideration the patient's young age and thus fertility, and she responded well to the therapy.

In conclusion, in patients recently diagnosed with GPA, it is important to consider the possibility of cardiac involvement and provide a thorough workup when cardiopulmonary symptoms are present. Cardiac MRI may be particularly useful when the initial presentation mimics that of an acute coronary syndrome, as it allows differentiating between acute myocarditis and manifestations of ischemia [9, 12].

## 5. Learning Points

- Granulomatosis with polyangiitis is a systemic vasculitis of the small- and medium-sized blood vessels characterized by granulomatous inflammation of both upper and lower respiratory tracts as well as necrotizing glomerulonephritis in the kidneys.
- Symptomatic cardiac involvement is rare and usually presents as pericarditis. However, coronary artery disease, myocarditis, valvulitis, and conduction abnormalities may be seen as well.
- Cardiac magnetic resonance imaging has emerged as a leading modality in the noninvasive diagnosis of myocarditis and is able to discriminate acute myocarditis from acute myocardial infarction.
